# Revising transcriptome assemblies with phylogenetic information

**DOI:** 10.1371/journal.pone.0244202

**Published:** 2021-01-12

**Authors:** August Guang, Mark Howison, Felipe Zapata, Charles Lawrence, Casey W. Dunn

**Affiliations:** 1 Center for Computational Biology of Human Disease, Brown University, Providence, RI, United States of America; 2 Center for Computation and Visualization, Brown University, Providence, RI, United States of America; 3 Research Improving People’s Lives, Providence, RI, United States of America; 4 Department of Ecology & Evolutionary Biology, University of California-Los Angeles, Los Angeles, CA, United States of America; 5 Department of Applied Mathematics, Brown University, Providence, RI, United States of America; 6 Department of Ecology & Evolutionary Biology, Yale University, New Haven, CT, United States of America; University of Western Sydney, AUSTRALIA

## Abstract

A common transcriptome assembly error is to mistake different transcripts of the same gene as transcripts from multiple closely related genes. This error is difficult to identify during assembly, but in a phylogenetic analysis such errors can be diagnosed from gene phylogenies where they appear as clades of tips from the same species with improbably short branch lengths. treeinform is a method that uses phylogenetic information across species to refine transcriptome assemblies within species. It identifies transcripts of the same gene that were incorrectly assigned to multiple genes and reassign them as transcripts of the same gene. The treeinform method is implemented in Agalma, available at https://bitbucket.org/caseywdunn/agalma, and the general approach is relevant in a variety of other contexts.

## Introduction

RNA-seq technology has made characterizing and quantifying transcripts practical and accessible for many researchers, providing novel insights into the study of gene evolution and function [1]. *De novo* transcriptome assembly tools in particular have become critical to many projects. These assemblers not only infer transcript sequences from raw reads, but also assess whether sets of similar transcripts should be assigned as splice variants of the same gene [2–4]. It can be difficult to distinguish whether differences between similar transcripts are due to technical variation (*i.e*., sequencing error), splice variation from the same gene, differences between alleles, or evolutionary divergence following gene duplication [5], especially as alternative splicing and gene duplication can have similar effects on transcript sequences [6, 7]. This challenge leads to transcript misassignment during transcriptome assembly. For example, different splice variants of the same gene are often inferred to be transcripts from different genes, inflating the number of inferred genes.

**Fig 1:**
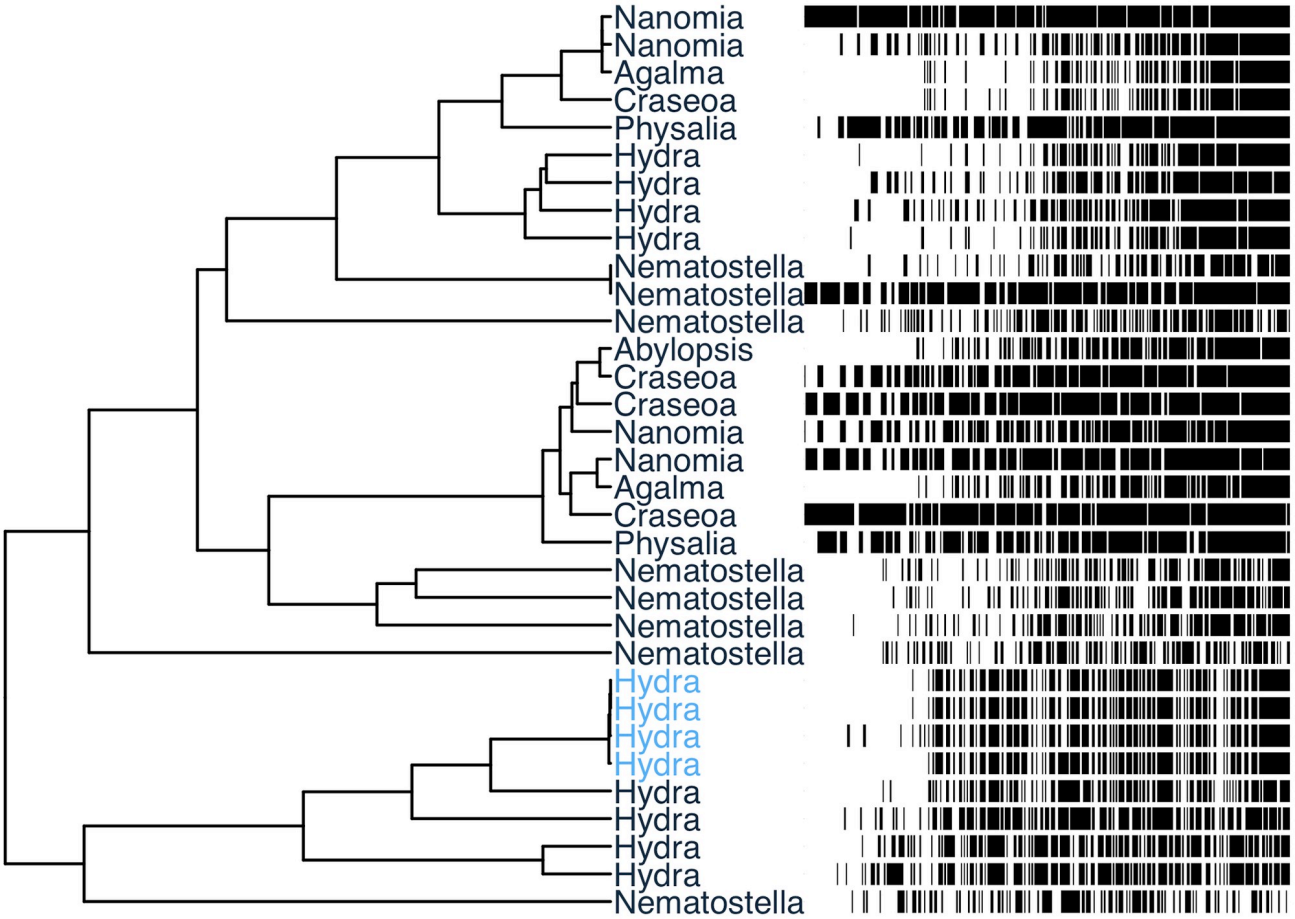
An example gene phylogeny from the test dataset before running treeinform. Each tip is an exemplar transcript that was initially assigned to a different gene. In front, corresponding multiple sequence alignment, with sites ordered from highest to lowest identity to the inferred ancestral site for clarity on sequence diversity. Black indicates a difference from the ancestral sequence. The four *Hydra* transcripts in color were assigned to different genes by Trinity [2] despite two of the transcripts sharing the exact same sequences, and the two other transcripts differing by a small gap. After treeinform, all transcripts from these four genes are reassigned to a single gene.

Transcript misassignment compromises downstream analyses like inferring accurate gene phylogenies or expression quantification as they assume error-free identification of genes and gene families [8]. While some tools exist to correct for assembly error in inferring certain gene history parameters such as gene duplication and loss [9], such tools generally do not update transcript assignments. Both transcript assignment and subsequent gene family (*i.e*., homolog) identification are based on sequence similarity as a proxy for evolutionary relatedness [10]. Thus, transcript assignment errors will appear in gene phylogenies as tips with improbably short branch lengths compared to theoretical models [11] (Fig 1). These tips can be deleted or masked, but that removes all trace of the misassigned transcripts, impacting expression quantification derived from mapping.

Here we propose a method, treeinform, to help identify transcript misassignment errors through subtree length thresholding at the gene tree level and correct such transcriptome assembly errors through revisiting and revising transcript assignments in the assembly. We show that after inferring gene phylogenies using the reassigned transcripts, we recover more accurate gene phylogenies as well as more accurate parameters describing gene history such as the number of duplication events. To test this method, we implemented it as a module within the phylogenomic workflow Agalma [12] available at http://bitbucket.org/caseywdunn/agalma.

## Materials and methods

### Overview of the approach

Phylogenetic analyses of transcriptome assemblies often proceed by first selecting a single exemplar transcript for each gene, such as the longest transcript or the transcript with the highest confidence [13]. Phylogenetic gene trees, which include data from multiple species and often multiple paralogs for some species, are then constructed. These gene trees are then analyzed in different ways depending on the intent of the study. For example, they are often then restricted to orthologs to examine relationships between species [14].

treeinform uses such gene phylogenies, where there is one exemplar sequence per gene but sometimes multiple putative paralogs (and therefore exemplars) per species. It flags tips from the same species that have improbably short branch lengths connecting them in such a gene tree. It then reassigns all the transcripts belonging to the putative genes that these exemplars represent to a single new gene, reselects an exemplar, and and rebuilds the gene tree. In many cases where the set of transcripts represented by an exemplar matter, such as when mapped reads are summed across transcripts to measure gene expression, this reassignment is preferable to the common practice of just dropping tips that are assessed to be assembly artifacts.

### Details of the algorithm and model

treeinform takes as input a set of gene phylogenies where each tip is a single exemplar transcript for a gene. It traverses each phylogeny estimating the total length (*i.e*., the sum of all branch lengths) of the subtree defined by each internal node. It then identifies subtrees with total length below a given threshold. If multiple tips (*i.e*., genes) belonging to a single species exist in an identified subtree, all transcripts for these multiple genes are flagged for reassignment to the same gene. *treeinform* outputs a list of transcripts for reassignment.

Before running treeinform, it is necessary to define a subtree length threshold. The default value for the threshold is 0.0005, determined by finding the intersection point of a mixture model [15] for branch lengths. Users may want to run their own analyses to determine an appropriate threshold by rerunning the Gibbs sampler for the mixture model, described in the Implementation section. To estimate the threshold, we use the relationship between subtree lengths and gene duplication history. When transcripts from the same gene are misassigned to different genes, gene trees/species tree reconciliation methods compensate by inferring additional duplication events [16]. Because misassigned transcripts have almost identical sequences, the inferred duplication events will be extremely shallow (*i.e*., closer to the tips rather than the root of the gene phylogenies), with correspondingly recent duplication times. Conversely, correctly assigned transcripts from different genes will largely have less similar sequences and thus older duplication times. Given a set of gene phylogenies and a species phylogeny, we implement a mixture model with two components as follows. One component models spurious duplication events, a second component models “true” duplication events [11], and a threshold such that subtree lengths less than the threshold have a probability > 95% of being spurious duplication events.

This mixture model implies that two different processes are operating simultaneously to generate the observed pattern of subtree lengths, one for the misassigned transcripts and one for the correctly assigned transcripts. To capture this pattern, we apply a mixture model to the inferred duplication times (equivalent to branch lengths) from the gene phylogenies. One component models duplication events and associated times arising from transcripts assigned to different genes that belong to the same gene (i.e., misassigned transcripts) and the other component models duplication events and associated times arising from transcripts assigned to different genes that in fact belong to different genes (*i.e*., correctly assigned transcripts).

We expect the implied duplication events of transcripts of the same gene that are misassigned to different genes to have extremely short duplication times approaching 0, and thus we model that component (Component 1) as a gamma distribution with parameters shape = *α* and rate = *β*. To model duplication events and associated times arising from the correctly assigned transcripts (Component 2), we use a constant rate birth-death process [11], which is well studied and often applied to gene analyses of duplication and loss. The probability distribution function in the birth-death model we use has parameters birth rate λ, death rate *μ*, and time of origin *t*_*or*_. Because we fit a time-calibrated phylogeny (*chronogram* with time of origin 1 onto the gene phylogenies *G* = {*G*_1_, *G*_2_, …, *G*_*K*_}, we made the assumption that all gene phylogenies times of origin *t*_*or*_ = 1. Some gene phylogenies can have duplication events predating the first speciation event, thus when we fitted chronograms onto those gene phylogenies they had times of origin greater than 1. We filtered these gene phylogenies of the mixture model and subsequent analyses.

Let *x*_*i*,*k*_ represent duplication time *i* from gene phylogeny *G*_*k*_, with *z*_*i*_ ∈ {1, 2} representing whether *x*_*i*,*k*_ is drawn from the 1st component (*z*_*i*_ = 1) or the 2nd component (*z*_*i*_ = 2). Then if *π*_1_ and *π*_2_ denote the overall probability that a duplication time belongs to the 1st and 2nd component respectively, Γ(*x*_*i*,*k*_|*α*, *β*) is the probability density function for the gamma distribution, and *f*(*x*_*i*,*k*_|*t*_*or*,*k*_ = 1, λ, *μ*) is the, we get the expression

$$\begin{array}{c} P\left(x_{i,k}\right)=\pi_{1}\Gamma \left(x_{i,k}|\alpha ,\beta \right)+\pi_{2}f\left(x_{i,k}|t_{or,k}=t,\lambda ,\mu \right) \end{array}$$

The posterior probability that a duplication time *x* is drawn from component 1 or component 2, *i.e*,. *P*(*z*|*x*) gives us a way to determine the probability of error. It can be inferred from Gibbs sampling as well, although it can also be estimated from the parameters of the mixture model. If we decide *x* is drawn from the 2nd component, then *P*(*z* = 1|*x*) will be the error probability, and if we decide *x* is drawn from the 1st component, then *P*(*z* = 2|*x*) is the error probability. If we care more about having fewer correctly assigned transcripts being erroneously flagged as misassigned, then we can use the posterior probability to select an appropriate threshold for treeinform by selecting *T* such that *P*(*z* = 2|*x*) < *α* for all *x* < *T*, where *α* is the error rate. In Bayesian decision theory this is equivalent to a loss matrix of $\left[\begin{array}{cc} \lambda_{11} & \lambda_{12}\\ \lambda_{21} & \lambda_{22} \end{array}\right]$, where each entry λ_*mn*_ is the penalty for selecting component *n* when *x*_*i*,*k*_ is actually drawn from component *m*.

From the threshold, we can back-calibrate to determine a subtree branch length threshold for use in treeinform. Specifically, we can take all duplication events with times below the intersection point on all chronogram-fitted gene phylogenies, map them to the equivalent events on the phyldog-outputted gene phylogenies, compute the subtree length of all events, and then take the maximum of those subtree lengths.

### Implementation

We implemented treeinform as a module within the end-to-end phylogenomic workflow Agalma [12]. The end-user needs to run Agalma once to generate a set of transcripts and gene phylogenies, which become the input to treeinform. The output of treeinform is a a list of transcripts for reassignment, which then become input for a second run of Agalma starting from RSEMEval [17].

We used Just Another Gibbs Sampler (JAGS) [18] to perform Bayesian Gibbs sampling [19] in order to infer the parameters in the mixture model: *α*, *β*, λ, and *μ* and the mixing proportions *π*_1_ and *π*_2_. This gave us the parameter estimates in Table 1.

**Table 1:** Summary of parameter estimates from JAGS.

	Lower95	Mean	Upper95	MCerr
*α*	0.2418870	0.2548908	0.2628810	0.0010395
*β*	1.7442500	1.9488374	2.1686600	0.0147482
*μ*	0.0000009	0.0119074	0.0356818	0.0001564
λ	2.7344800	2.8621597	2.9990300	0.0008479
*π*_1_	0.3167680	0.3386287	0.3604700	0.0001405
*π*_2_	0.6395300	0.6613713	0.6832320	0.0001405

We decided to use *α* = 0.05 as the significance level. This gives us the loss matrix $\left[\begin{array}{cc} 0 & 1\\ 19 & 0 \end{array}\right]$ and the intersection point 0.0003255. Backcalibrating from the intersection gives us a threshold of 0.000562, which we approximate with 0.0005.

## Results

All analyses can be reproduced with code from https://github.com/caseywdunn/ms_treeinform.

### Comparing transcript clustering methods on *Siphonophora*

**Fig 2:**
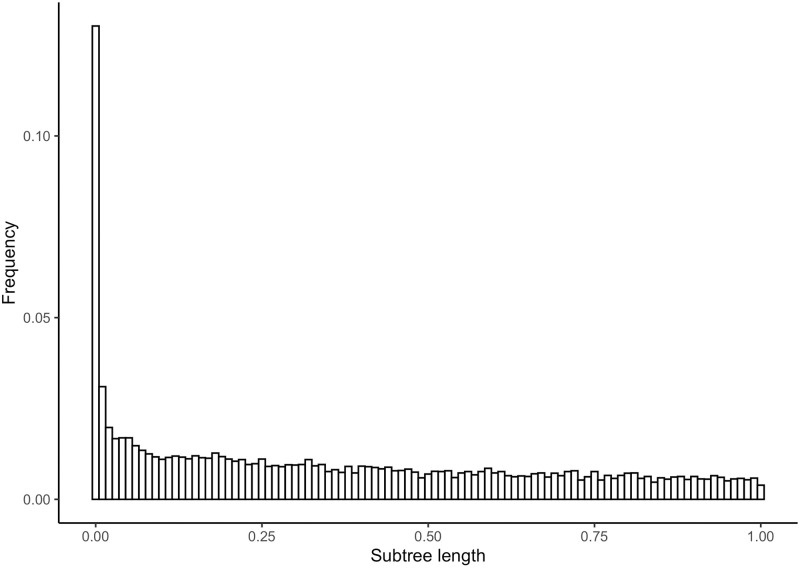
Histogram of subtree lengths for internal nodes in each gene phylogeny from the test dataset containing tip descendants from the same species. Subtree lengths greater than 1 were filtered out for clarity.

We initially assessed how widespread the problem of transcript misassignment is on a test dataset from a broader phylogenomic study of *Siphonophora* (Cnidaria) [20]. By default, Agalma uses the popular transcriptome assembler Trinity [2]. This data set included 5304 gene phylogenies. As in any comparative genomics study, tips in each gene phylogeny correspond to exemplar genes *i.e*., an exemplar transcript per gene). For each node in each of the gene phylogenies, we calculated the total length of the corresponding subtree. This is the sum of the length of all branches in the subtree defined by the node. Because alternative transcripts of the same gene largely have very similar sequences, an excess of short subtrees would be a strong indication of transcript misassignment. This is the pattern we recovered (Fig 2).

**Fig 3:**
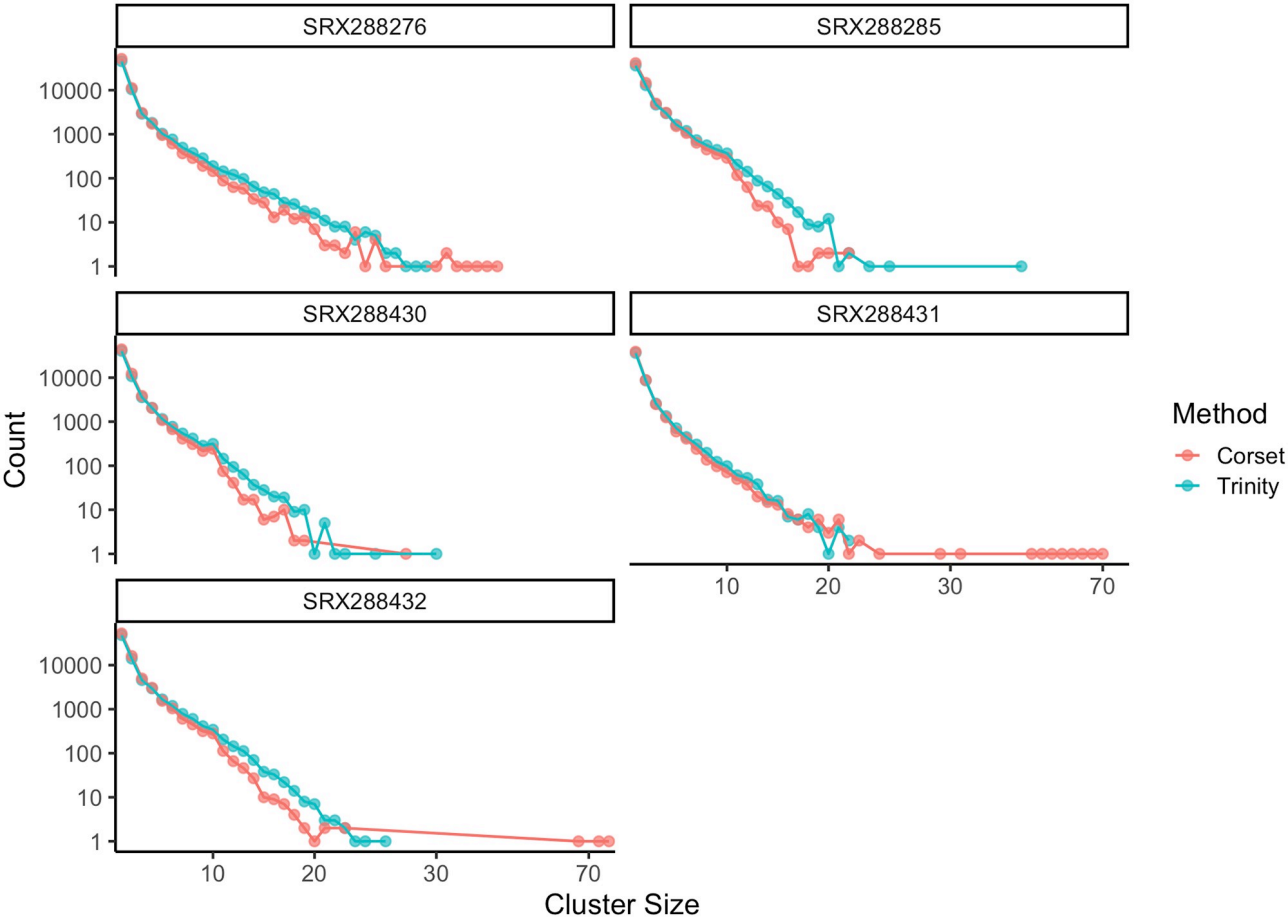
Cluster size counts for Trinity assembly and Corset clustering algorithm on Trinity contigs. There are 3 Trinity clusters with size greater than 30, while there are 20 Corset clusters with size greater than 30.

To assess whether the transcript misassignment errors were localized to Trinity or are a more general problem to transcriptome assembly, we compared Trinity transcript clustering results with another transcript clustering tool, Corset [21] for the 5 species that had to be assembled. For 3 of the samples (SRX288285, SRX288430, SRX288431) we also ran cd-hit [22] to remove transcripts with 100% identity in order to address some speed issues in Corset. The distribution of cluster sizes (Fig 3) suggests that Corset tends to overcluster compared to Trinity, which would lead to similar misassignment errors.

Additionally, we computed the Adjusted Rand Index (ARI) [23] to get a sense of the similarity between the Trinity and Corset clusterings. (Table 2) The Adjusted Rand Index computes the proportion of pairs that either both belong to the same cluster, or that both belong to different clusters, corrected for random assignment chance. The ARI ranges from 0 to 1, with 0 meaning the clusterings are maximally dissimilar and 1 meaning that the clusterings are exactly the same. Though we have no ground truth, the ARI suggests that Trinity and Corset clusterings are more similar than dissimilar, and in the case of SRX288285, SRX288430, and SRX288431 are extremely similar.

**Table 2:** Adjusted Rand Index between Trinity and Corset clusterings by sample.

Sample	Adjusted.Rand.Index
SRX288276	0.5805402
SRX288285	0.8353263
SRX288430	0.8094703
SRX288431	0.8089121
SRX288432	0.7835530

**Fig 4:**
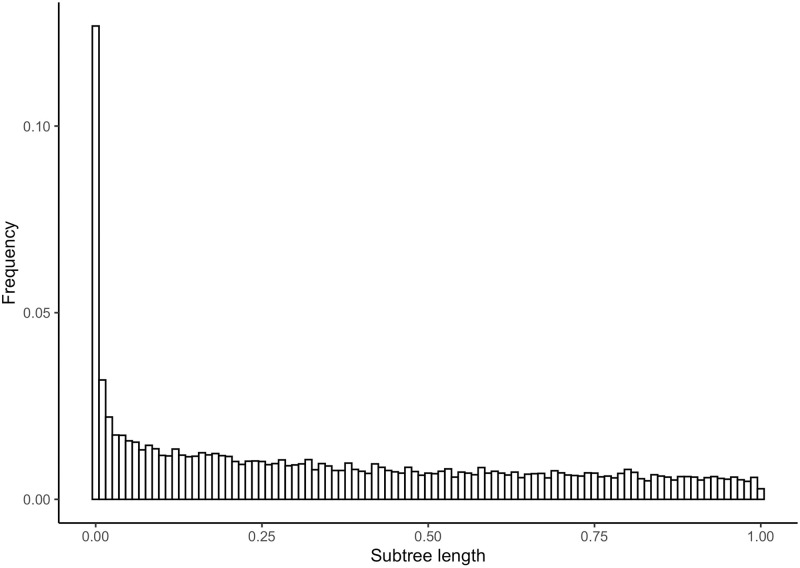
Histogram of subtree lengths for internal nodes in each Siphonophora subset gene tree from Agalma with Corset clusterings containing tip descendants from the same species. Subtree lengths greater than 1 were filtered out for clarity.

Fig 4 shows the histogram of subtree lengths for internal nodes in each *Siphonophora* subset gene phylogeny with Corset clusterings. The distribution of subtree lengths for Corset is not different from the distribution of subtree lengths for Trinity (two-sample Kolmogorov-Smirnov test [24], D = 0.0104) It shares the same shape and scale as in Fig 2, and the distribution of subtree lengths for Corset is not different from the distribution of subtree lengths for Trinity (two-sample Kolmogorov-Smirnov D = 0.0104). This indicates that regardless of transcript clustering method, transcript misassignment errors persist. Given the intrinsic challenges of correctly assigning transcripts to genes it is likely that misassignment errors is a pervasive problem for other transcriptome assemblers.

### Validating the effectiveness of treeinform on *Siphonophora*

**Fig 5:**
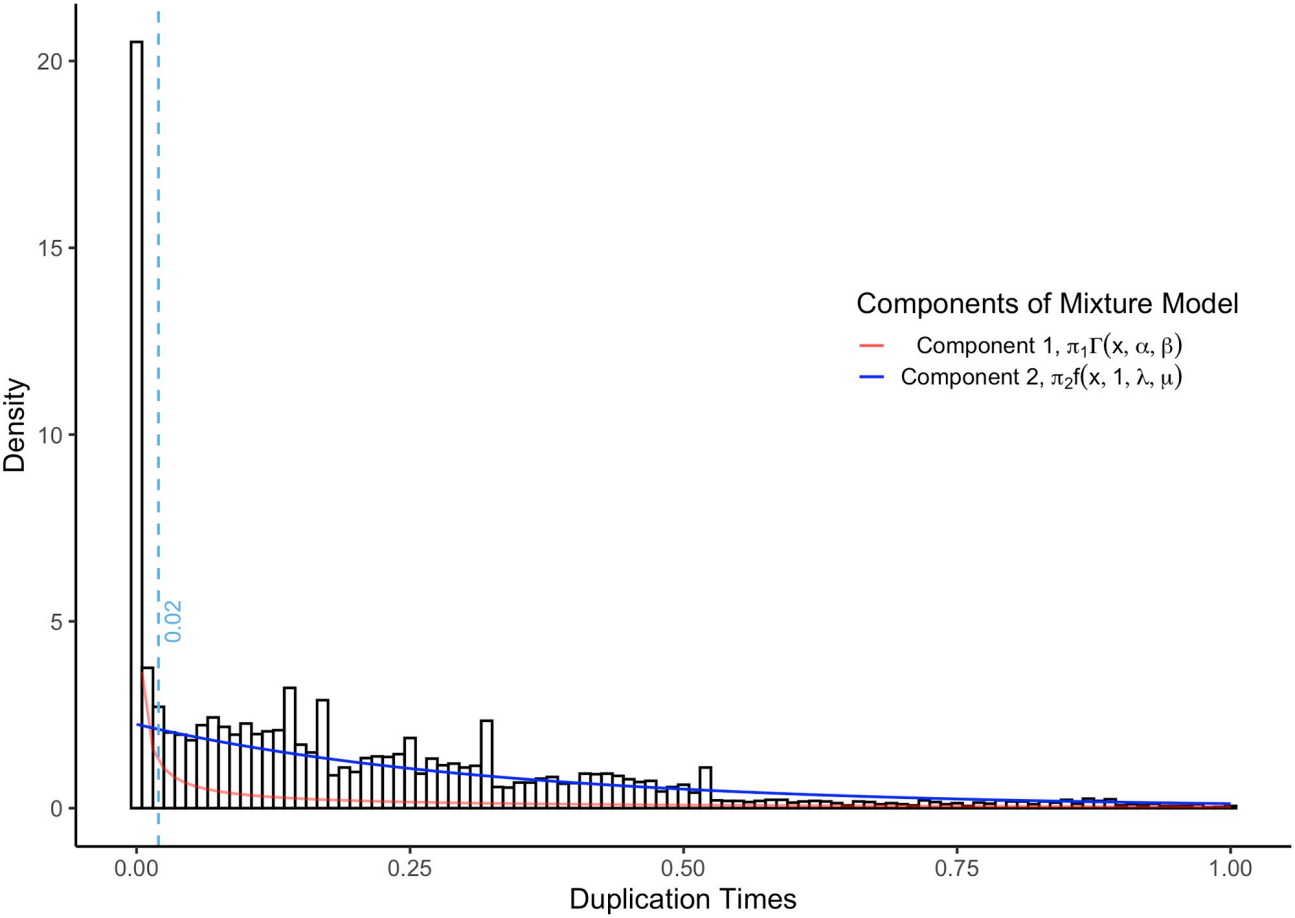
Histogram of the inferred duplication times with an overlaid mixture model. Component 1 of the mixture model (red) captures the technical issues we address here, where transcripts from the same gene are assigned to different genes, and component 2 (blue) captures the true biological pattern, where transcripts from different genes are correctly assigned to different genes. We first ran phyldog [16] on the test dataset using the multiple sequence alignments and a given species phylogeny [20]. This provided gene phylogenies with internal nodes annotated as duplication or speciation events. We then used the annotations to time-calibrate the gene phylogenies for the mixture model.

We took three different approaches to assess the efficacy of treeinform. First, we spot checked the results to confirm that they were biologically and technically sensible. This provided detailed confirmation on a small fraction of the output. Second, we compared duplication time distributions of the entire input and output tree sets against theoretical expectations [11] (Fig 5). To compare these distributions, we used the Kullback-Leibler (KL) distance, or relative entropy, an information theoretic approach to measures the distance between two distributions [25]. Duplication time distributions of output tree sets under the default threshold had a lower KL distance to theoretical expectations than the input tree sets (Table 3). This provided an assessment of the impact of the method across the entire output.

**Table 3:** Kullback-Leibler distances between duplication times after running treeinform with different thresholds and theoretical duplication times.

	KL.Distance
Before	0.2543703
0.07	0.1395099
0.05	0.1101704
0.005	0.0946684
0.0005	0.0997587
5e-05	0.1013232

**Fig 6:**
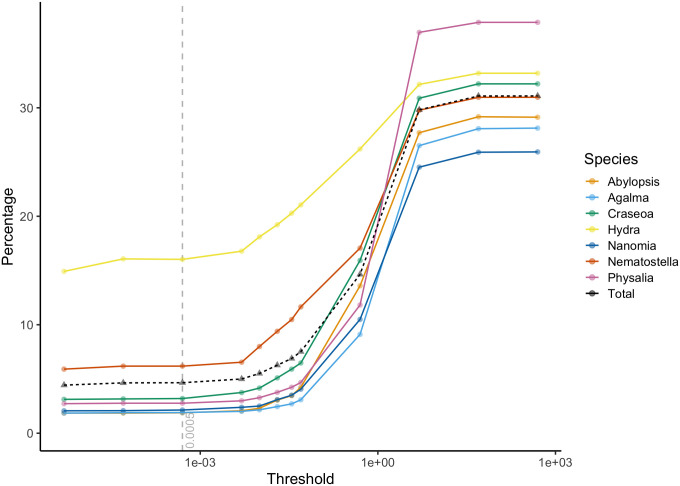
Percentage of reassigned transcripts (log scale). 47,688 genes were included in the gene phylogenies, of which 23,396 (49.06%) were in gene families of 2 or more, and thus candidates for reassignment. The default threshold for treeinform is marked by the grey vertical dashed line.

In order to validate that treeinform improved the accuracy of assigning transcripts to genes under the specified threshold, we performed two analyses. First, we plotted the percentage of reassigned genes at different thresholds to assess the performance of the default threshold value of 0.0005 (Fig 6). The percentage of reassigned genes begins to plateau below the default threshold. In contrast, the percentage of reassigned genes increases very quickly above the default threshold, increasing the likelihood of treeinform to reassign transcripts from different genes to the same gene.

We also looked at the percentage of reassigned genes for each species to assess how variable transcript misassignment was by species. This percentage was variable, with *Hydra magnipapillata* having a much higher proportion of reassigned genes (16.03%) at the threshold. This affected the total proportion of reassigned genes, with the majority (46-47%) of reassigned genes at and around the treeinform threshold coming from *Hydra magnapapillata*. For the remaining species, 1.88-6.18% of genes were reassigned at the default threshold.

**Fig 7:**
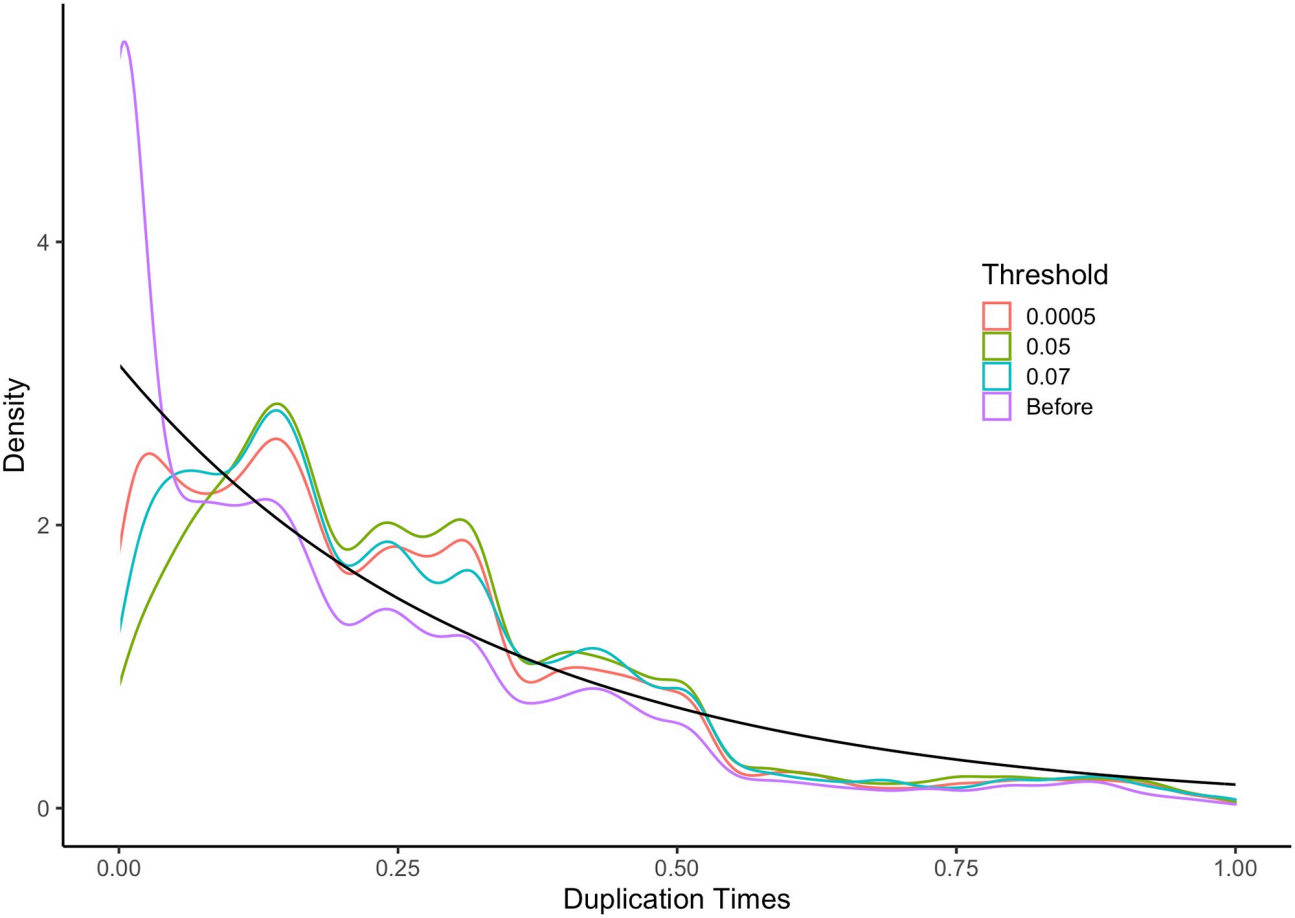
Density from theoretical and the empirical density under 3 different thresholds before treeinform was run. The distribution before treeinform has a large peak on the left that is removed by treeinform with all examined thresholds. Black line represents theoretical density.

Second, we compared the density of duplication times under the model provided for Component 2 of the mixture model to the distribution of estimated duplication times for gene trees from Agalma before and after treeinform under 3 different thresholds: 0.05, 0.0005, and 0.07 (Fig 7). We fitted chronograms onto all gene phylogenies from Agalma and filtered out those gene trees with time of origin greater than 1, so that duplication times were comparable between trees. Visually, the analyses with the 0.0005 threshold comes closest to the theoretical. However, we note that in all cases ridges exist in the empirical densities that are not present in the theoretical density of duplication times. We suspect this is due to speciation events that occur after duplication events, representing another branching process that is not reflected in the constant rate birth-death process we use as our model for Component 2.

We computed the Kullback-Leibler distance (KL) [25] between the distributions of duplication times under different thresholds and the theoretical distribution of duplication times (Table 3). The KL distance between the distribution of duplication times after running treeinform remains about the same below 0.005. This indicates that treeinform produces more accurate gene trees with appropriate threshold selection.

### Evaluation on other taxa

**Fig 8:**
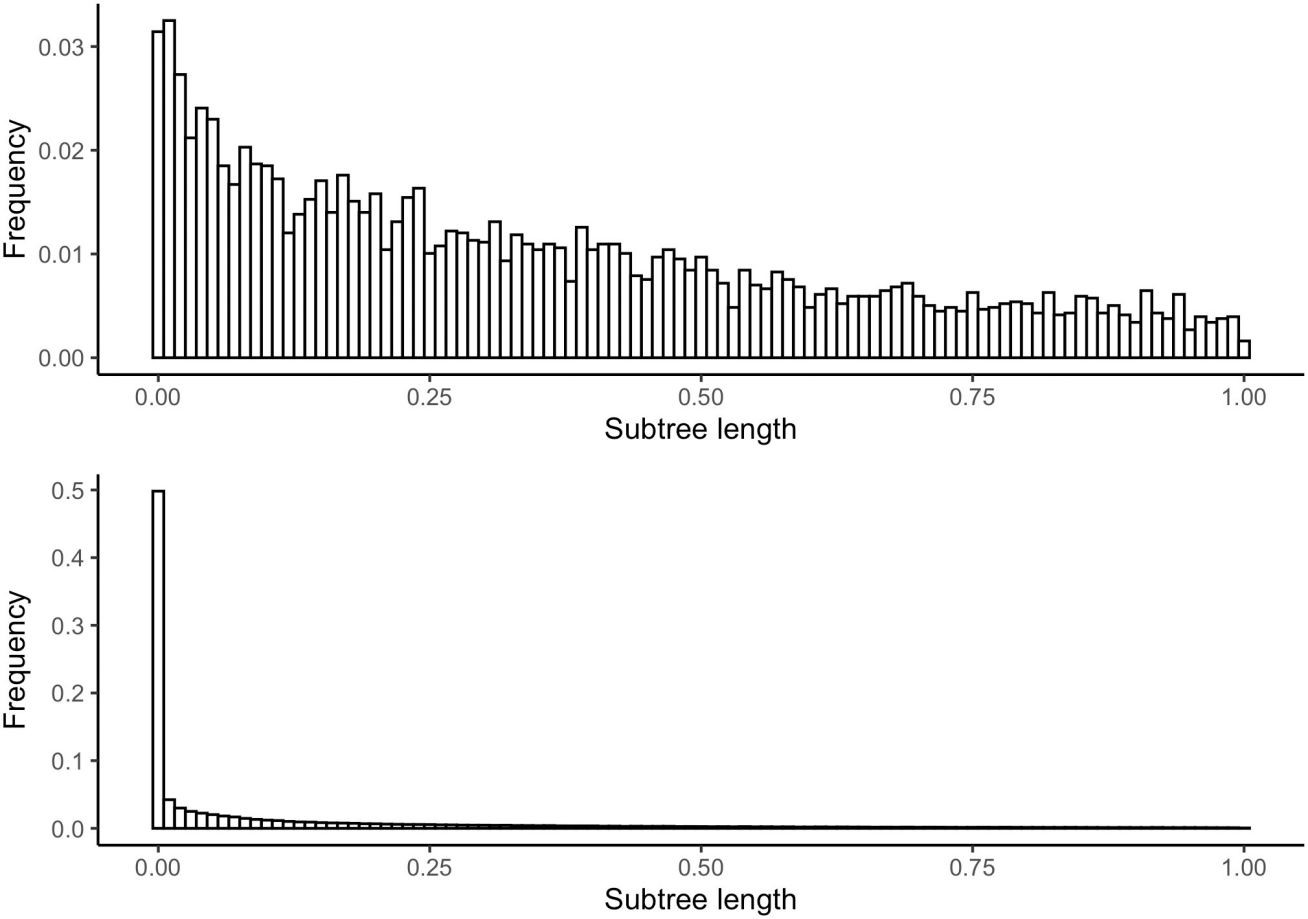
Histogram of subtree lengths for internal nodes in each *Drosophila* and *Echinoidea* subset gene tree from Agalma containing tip descendants from the same species. Top is *Drosophila* and bottom is *Echinoidea*. Subtree lengths greater than 1 were filtered out for clarity.

In addition to evaluating treeinform on the *Siphonophora* data set, we also evaluated treeinform on two phylogenies with publicly available sequencing data, *Drosophila* [26] and *Echinoidea* [27], each containing model species (*Drosophila ananassae, Drosophila melanogaster, Drosophila pseudoobscura, Drosophila simulans, Drosophil virilis* and *Strongylocentrotus purpuratus*, respectively) with different degrees of transcript quality and genetic diversity. Because the model species have associated known coding sequences (CDS), we were able to assess transcript assignment accuracy both pre and post-treeinform. To do this, we ran Trinity on the raw reads, selected representative transcripts from the assigned genes using RSEM-Eval, and then ran BLAST [28] with the representative transcripts from each genes against the available CDS. We then ran treeinform. After running treeinform, a representative transcript was selected from the reassigned genes using RSEM-Eval, and compared to the available CDS using BLAST. We took pairs of representative transcripts and computed whether they were correctly assigned to the same gene, incorrectly assigned to different gene clusters, or incorrectly assigned to the same gene cluster. We did this for multiple treeinform thresholds. For *Drosophila*, less than 1% of tips were reassigned by treeinform even at thresholds as high as 0.5, suggesting that the Trinity assembly and associated filtering heuristics for downstream analyses are quite accurate. For *Echinoidea*, around 5-10% of tips were reassigned by treeinform at various thresholds, in line with what we saw for the *Siphonophora* dataset. This discrepancy is also reflected in the respective histograms of their subtree lengths (Fig 8), with a large peak of subtrees with length close to 0 present only in *Echinoidea*.

**Fig 9:**
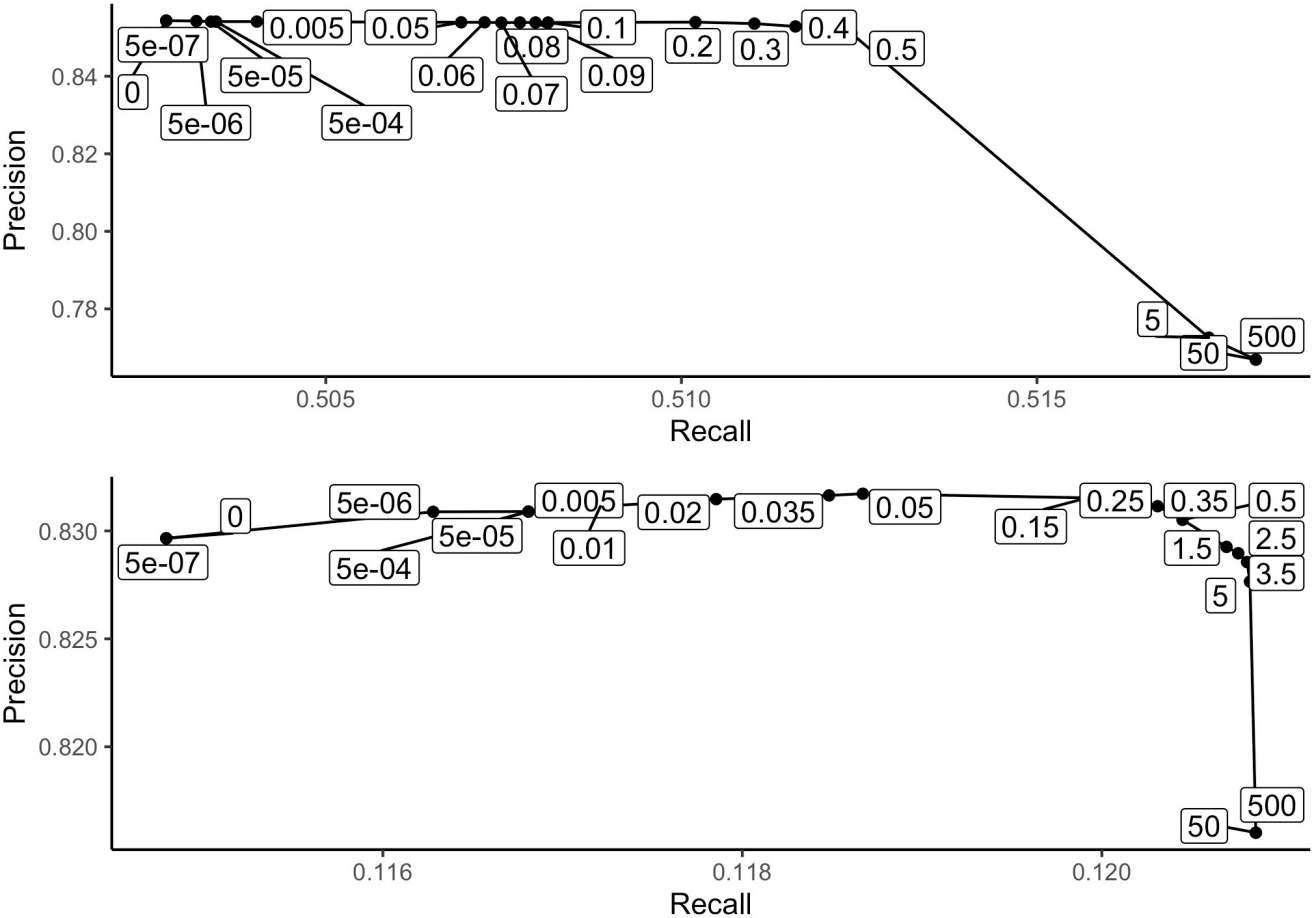
Precision *vs*. recall for pairs of transcripts with regards to known CDS as treeinform threshold increases. Top: Plot for *Drosophila* with CDS. Precision does not increase with any threshold, only recall. Bottom: Plot for *Strongylocentrotus purpuratus*. The biggest improvement is made at a threshold value 5e-06, with precision and recall both increasing up to a threshold of 0.05.

As the treeinform threshold increases, we expect that while the number of transcript pairs correctly assigned to the same gene cluster will increase, the number of pairs incorrectly assigned to the same gene cluster will also increase. This can be examined through comparing precision and recall, with true positives defined as the number of transcript pairs correctly assigned to the same gene cluster, false positives defined as the number of transcript pairs incorrectly assigned to the same gene, and false negatives defined as the number of transcript pairs incorrectly assigned to different genes. Although our expectations appear to hold, it is not a linear relationship, and we see that at smaller thresholds immediate improvements in transcript assignment can be made without creating a lot of erroneous assignments (Fig 9). For *Drosophila* only recall increases while precision remains flat, indicating that treeinform does not have much of an effect either negatively or positively, while for *Strongylocentrotus purpuratus*, both precision and recall increased up to a threshold of 0.05, indicating that treeinform is correctly assigning transcript pairs to the same gene without making many incorrect assignments. The benefit of using the mixture model is the ability to select an exact threshold for maximizing correct reassignment and minimizing additional errors.

### Discussion

The central goal of transcriptome assembly is to use sequence reads to estimate the correct sequence of the original transcripts, and then to assign these transcripts to their respective genes. One of the biggest challenges in transcriptome assembly is to identify whether the sequence variance of transcripts is due to technical factors (such as errors introduced in library preparation and sequencing), splicing differences, different alleles of the same locus, or evolutionary divergence between closely related duplicate genes. It can be difficult to determine if slightly different transcripts are variants of the same gene or are derived from different closely related genes. This has the potential to compromise downstream analyses such as phylogenetic gene tree estimation or gene expression quantification [29].

We looked at the prevalence of transcript misassignment in *Siphonophora* by looking at subtree branch length distributions and found far more subtrees with branch length sums close to 0 than we would expect. We found from spot checking gene trees and alignments that many of the genes appeared to be misassigned, with branch lengths of almost 0 and identical sequences. Through our algorithm treeinform which flags and reassigns transcripts with subtree lengths under a model-determined threshold, we were able to bring the observed branch length distribution closer to the expected distribution in gene trees, and improve transcript clustering accuracy in one system with known transcript-gene mappings.

Despite our algorithm reducing the prevalence of misassigned transcripts, it has variable results across different organisms. The KL divergence to the expected theoretical duplication-loss distribution was decreased by at least 0.15 in *Siphonophora*, and both precision and recall for transcript clustering increased in *S. Purpuratus*, but only recall increased in *Drosophila*. Additionally, the subtree branch length histogram for *Drosophila* without treeinform did not feature a large peak close to 0, unlike for *Siphonophora* and *Echinoidea*. (Fig 8) One possible reason for this is that *Drosophila* is a well-characterized model organism and assembly algorithms such as Trinity are trained on and optimized for this kind of organism. Another possibility is differences in genetic diversity [30], with clades like *Siphonophora* and *Echinoidea* posing more difficulty for assemblers due to higher genetic diversity, and thus treeinform having more of an impact. Future work could include looking into the relationship between genetic diversity and treeinform capabilities.

In general, we expect that due to variation in rates of taxon and gene evolution the algorithm may not improve much on the original results. The subtree branch length plots (Figs 2, 4 and 8) serve as a useful diagnostic tool to tell us if the algorithm will work well or not. When no significant peak exists in the subtree branch length plot, that suggests that errors in assignment of transcripts from different genes to the same gene are few. In that case the algorithm is not necessarily needed, and in addition the components that comprise the mixture will not be well-separated [31]. One way to confirm this is to simulate both components given parameters indicating low transcript misassignment rates and high transcript misassignment rates, and seeing if the mixtures are well-separated or not.

Additionally, the selection of a threshold based on the mixture model intersection could be improved in the future. First, a singular threshold where all tips with subtree branch length under that threshold are reassigned ignores the possibility that some tips with branch lengths close to 0 may actually be from extremely recent duplication events. Since the mixture model assigns probabilities to duplication times being spurious or true, a simple modification to our approach could be to treat reassignment stochastically according to mixture model probabilities, rather than use a single threshold. The mixture model itself could also incorporate information besides only the duplication times. Alignment information in the form of profile hidden Markov models [32] could be incorporated into the mixture model, or more specific features from alignment or sequence-based tools for distinguishing between transcripts from the same gene and transcripts from different genes [21, 33].

Although other approaches to distinguishing transcripts from the same gene and transcripts from different genes exist, treeinform takes advantage of gene information across species and the Markovian dependency assumption in phylogenetic workflows [8], as not only can inferences about the gene trees be made solely from the gene assemblies, but inferences about the gene assemblies can be made solely from the gene trees as well. Using phylogenetic information, our new approach reassigns transcripts to their corresponding gene when different transcripts of the same gene are mistaken as transcripts from different closely related genes. Analyses of treeinform shows that it brings estimates of duplication times much closer to theoretical expectations. treeinform has been applied to multiple phylotranscriptomic studies [20, 27] as part of Agalma1.0, and will be useful for any studies requiring accurate gene trees, in particular accurate counts of different genes in expression studies.
